# Supplementary figures and images for: The prophylactic value of TNF-α inhibitors against retinal cell apoptosis and optic nerve axon loss after corneal surgery or trauma
Source: Acta Ophthalmol. Author manuscript; Available in PMC 2024 May 1. (PMC10997738; doi:10.1111/aos.15786)

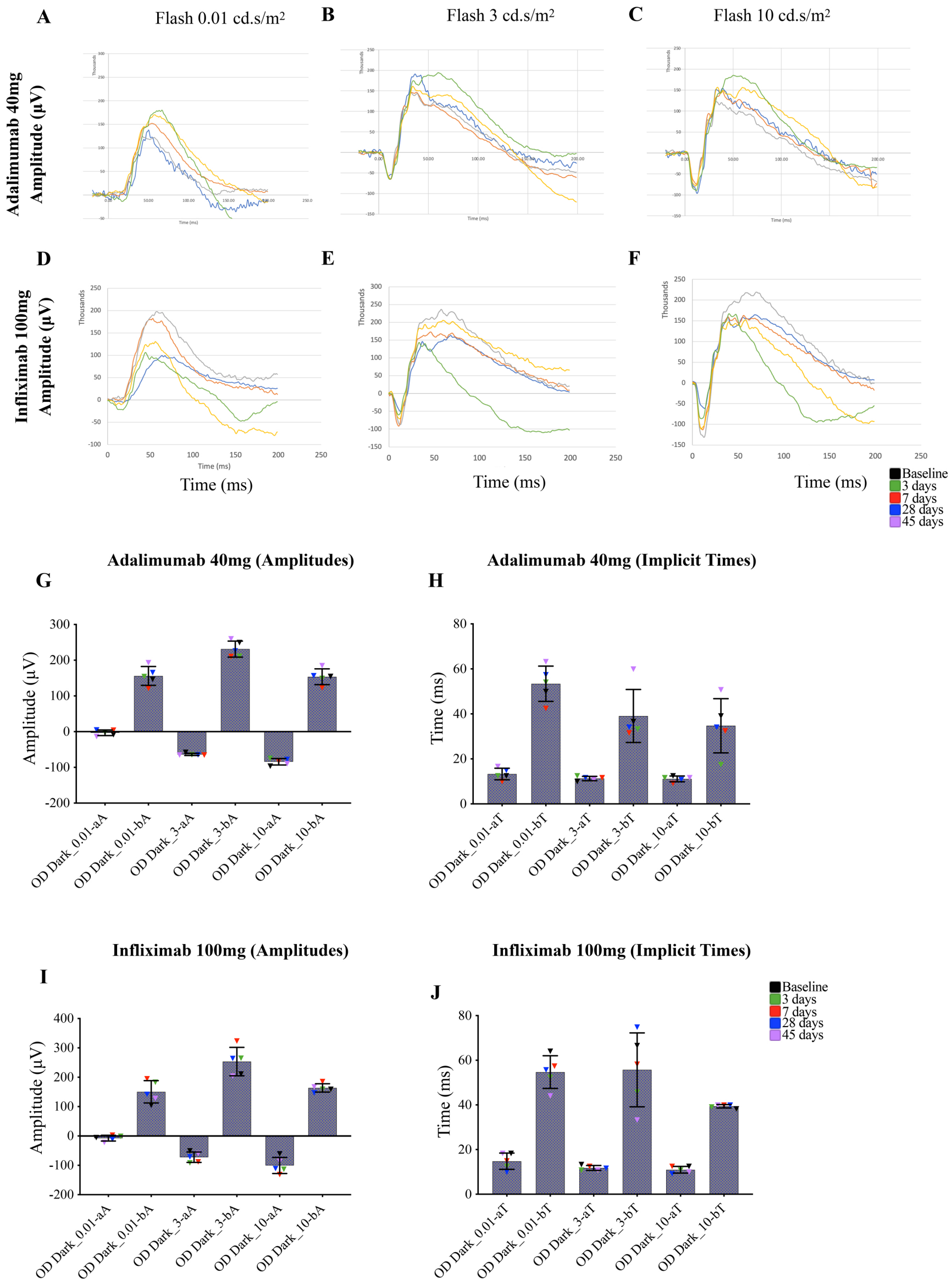

Supplement: Suppl Fig 1 [file NIHMS1950771-supplement-Suppl_Fig_1.pdf]

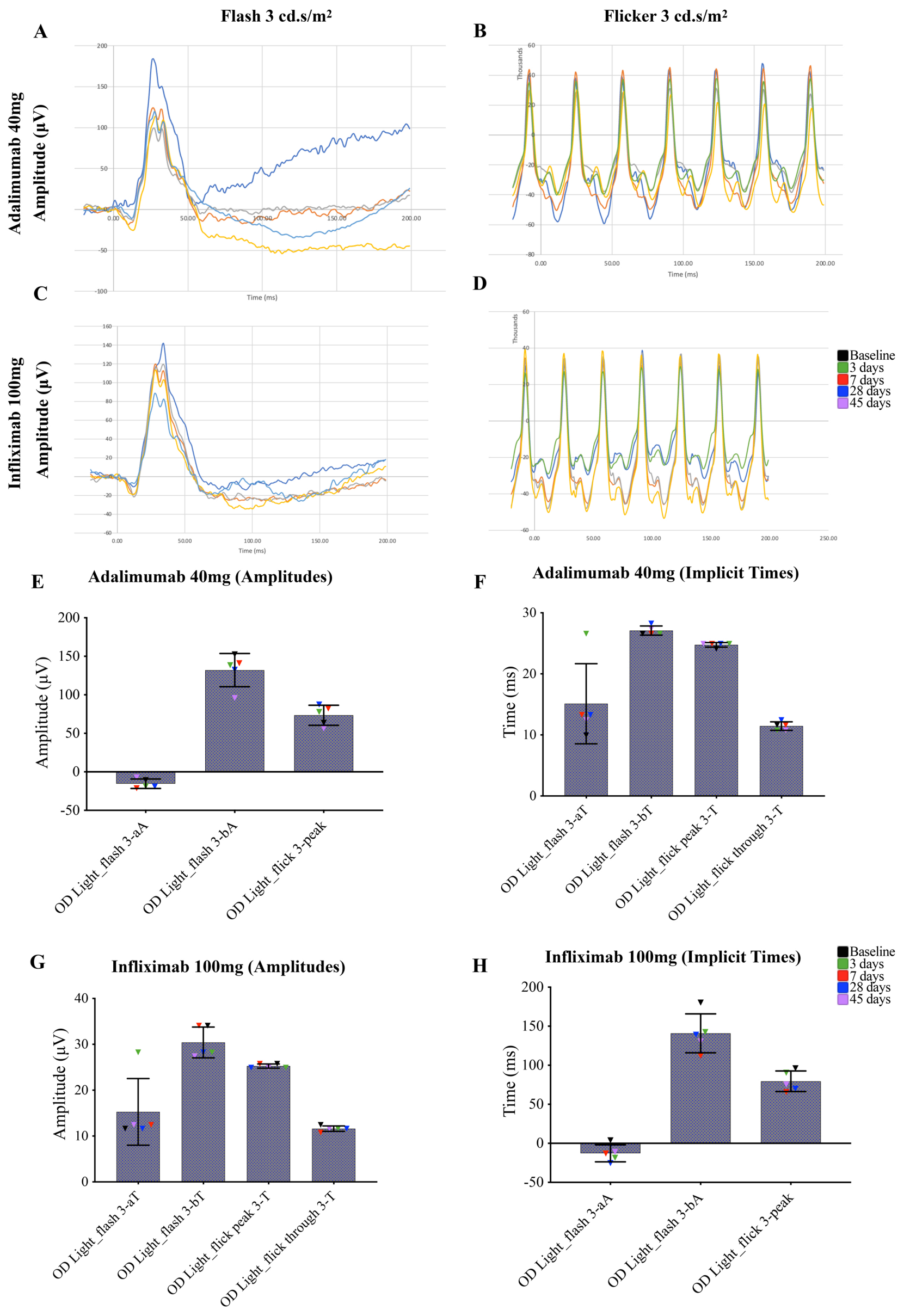

Supplement: Suppl FIg 2 [file NIHMS1950771-supplement-Suppl_FIg_2.pdf]

### Adalimumab 40mg SC (superior retina)

Baseline

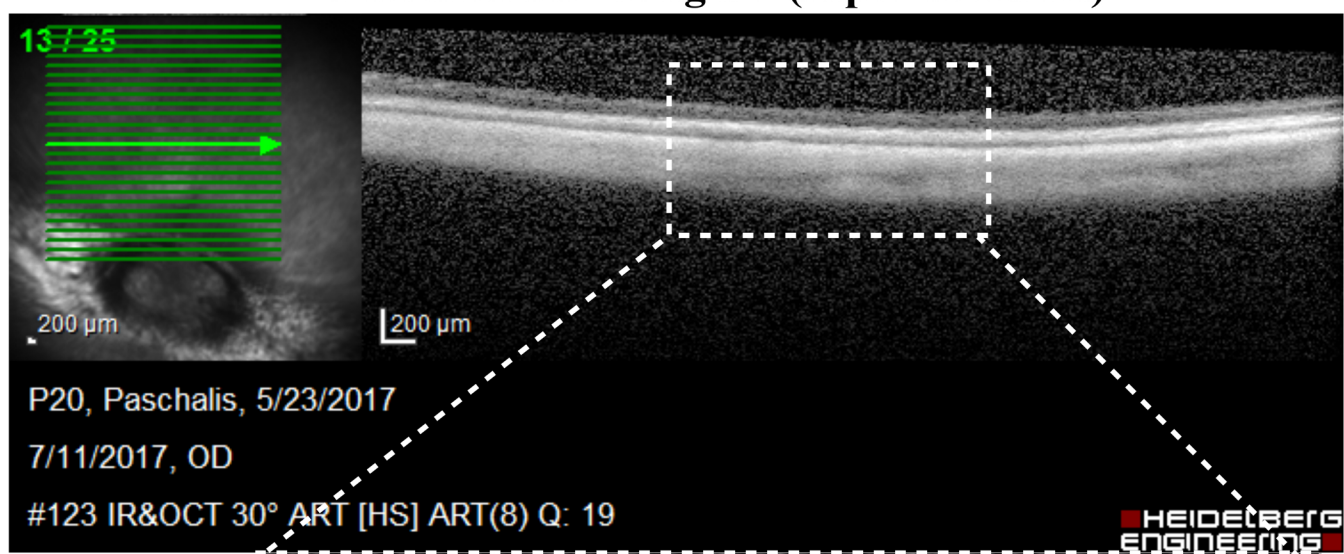

Baseline

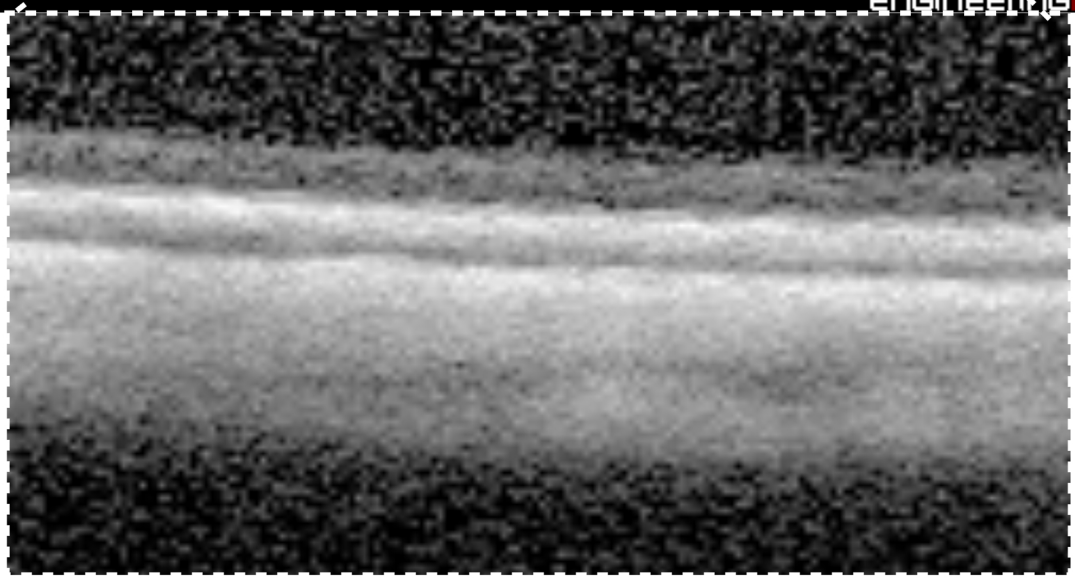

50 days post-  
injection

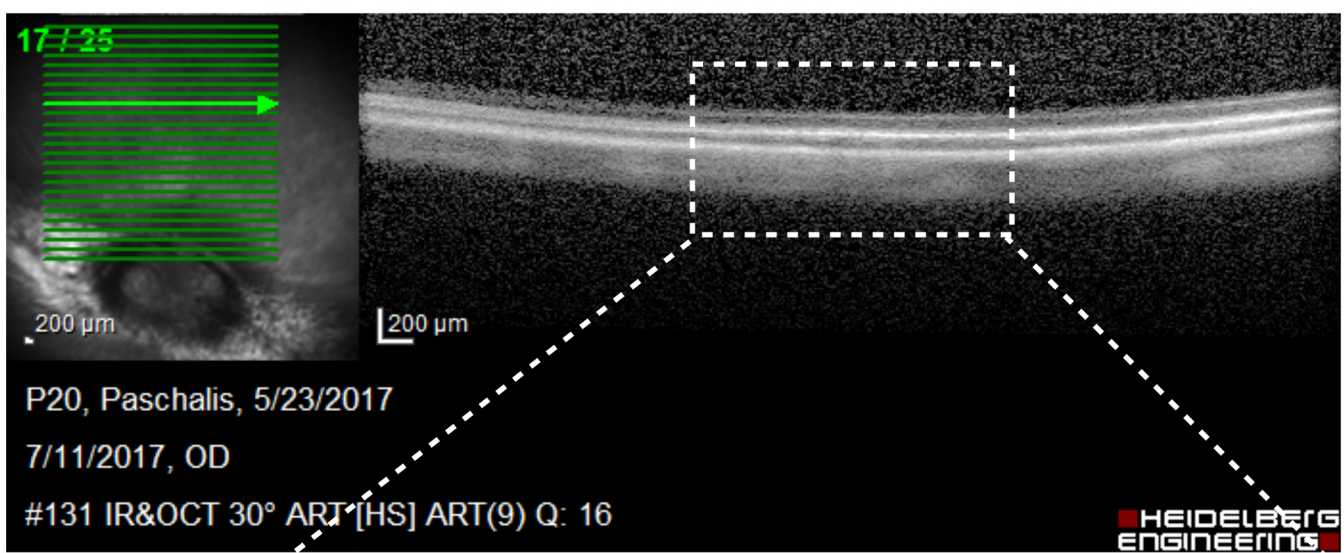

50 days post-  
injection

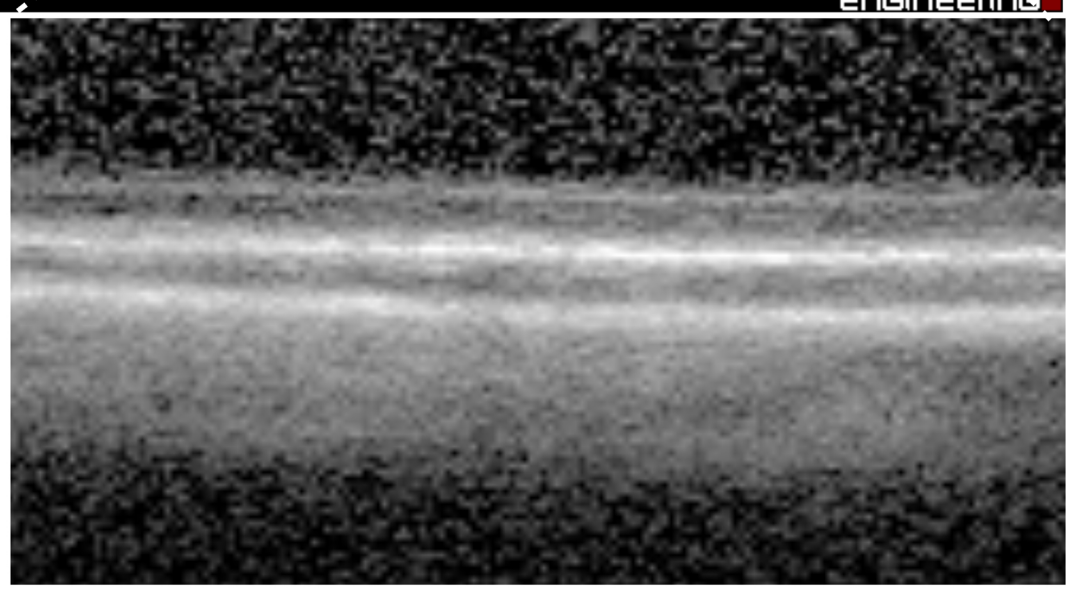

Supplement: Suppl Fig 3 [file NIHMS1950771-supplement-Suppl_Fig_3.pdf]
