# Supplementary material for: The prophylactic value of TNF-α inhibitors against retinal cell apoptosis and optic nerve axon loss after corneal surgery or trauma
Source: Acta Ophthalmol. Author manuscript; Available in PMC 2024 May 1. (PMC10997738; doi:10.1111/aos.15786)
Supplement: Suppl Fig Legends [file NIHMS1950771-supplement-Suppl_Fig_Legends.docx]

**Supplemental Figure 1**. **Toxicity: Dark-adapted ERG assessment after subconjunctival injection of adalimumab and infliximab.**

Dark-adapted electroretinography (ERG) using 0.01, 3 and 10 cd.s/m^2^ light intensities after subconjunctival injection of **(A - C)** 40 mg adalimumab and **(D - F)** 100 mg infliximab. The contralateral eyes were used as internal controls, while saline (sham) injected eyes served as treatment controls. ERG quantification in 40 mg adalimumab injected eyes **(G)**Amplitudes **(H)** Implicit times. 100 mg infliximab injected eyes **(I)** Amplitudes and **(J)** Implicit times. Measurements were performed at baseline (7 days before injection) and 3, 7, 28, and 45 days after injection. **(G - J)** Quantification was performed for “a" and “b”-wave “A”mplitude and “T”ime responses at“0.01”, “3”, and “10” cd.s/m^2^ light intensities in injected eyes. High doses of subconjunctival injection of adalimumab and infliximab did not cause appreciable changes in the dark-adapted ERG responses, although optic nerve degeneration was evident at 40 mg of adalimumab with PPD staining. One animal per dose with serial measurements, total n=8.

**Supplemental Figure 2**. **Toxicity: Light-adapted ERG assessment after subconjunctival injection of adalimumab and infliximab**.

Light-adapted electroretinography (ERG) using 3 cd.s/m^2^ flash and flicker light stimulation after subconjunctival injection of **(A, B)** 40 mg adalimumab and **(C, D)** 100 mg infliximab. **(E, F)** Amplitudes **and** corresponding implicit times of ERG in 40 mg adalimumab and **(G, H)** 100 mg infliximab injected eyes. Measurements were performed at baseline (7 days before injection) and 3, 7, 28, and 45 days after injection. **(E - H)** Quantification was performed for “a" and “b”-wave “A” amplitude and “T” Time responses at “3” cd.s/m^2^ “flash” and “flicker” light intensity in injected eyes. High dose subconjunctival injection of adalimumab and infliximab did not cause appreciable changes in the light-adapted ERG responses. One animal per dose with serial measurements, total n=8.

**Supplemental Figure 3. Magnified optical coherent tomography of the retina 50 days after subconjunctival administration of adalimumab 40 mg.**

Representative high magnification*in vivo* optical coherence tomography (OCT) of the superior retinal quadrant at baseline and 50 days post subconjunctival injection with 40 mg adalimumab showing thinning of the neuroretina.
